# Acceptance of COVID-19 vaccination among parents of children with autism and other neurodevelopmental disorders in Saudi Arabia: a cross-sectional study

**DOI:** 10.1186/s12889-023-16127-3

**Published:** 2023-06-26

**Authors:** Ali Jawad Al Saad, Ghadeer Mohammed Alhassan, Maryam Saleh Albedaiwi, Fatimah Fathi Alqattan, Fatimah Ali Aleisa, Hawra Wasel Alabdulmuhsin

**Affiliations:** King Faisal University, Alahsa, Saudi Arabia

**Keywords:** Vaccine hesitancy, COVID-19, Autism, Neurodevelopmental disorders, Saudi Arabia

## Abstract

**Background:**

Acceptance of COVID-19 vaccination was noticed to be less common among parents of children with autism spectrum disorder (ASD) and other neurodevelopmental disorders. This study aimed to explore the beliefs and willingness of parents of children with neurodevelopmental disorders about COVID-19 vaccine and understand how certain factors influencing the vaccine decision-making process differ between them and other parents’ groups.

**Methods:**

A cross-sectional study was conducted between August to November 2021. An Arabic online survey was distributed in August 2021 to collect the study’s data. 400 parents from all the major regions in Saudi Arabia participated in and shared their beliefs about the new COVID-19 vaccination for their children.

**Results:**

Out of 400 participants, 381 of them were eligible to answer the survey (95.2%). The total number of parents of children with neurodevelopmental disorder was 158 (41.5%), was compared to responses of parents of heathy children 223 (58.5%). 85 (53.8%) of them were ready to vaccinate their children with COVID-19 vaccine. While 36 (22.8%) were hesitant, the rest 37 (23.4%) did not want to vaccinate their children at all. Only a small number 16 (10.1%) have beliefs of vaccines as a cause of their child’s neurodevelopmental disorder. A total of 79 out of 131 responses were received from both parents’ groups. Fear of long-term side-effects was the most common reason reported by 41 responders out of 64 (64.06%) from parents of healthy children and 38 responders out of 67 (56.71%) from parents of diagnosed children. Another reason reported by parents of younger children in both groups was the child’s age. Having a healthcare relative worker was significantly associated with the vaccine decision making (*p* < .001).

**Conclusion:**

The acceptance rate of COVID-19 vaccination of parents of children with neurodevelopmental disorders was low compared to the parents of healthy children in Saudi Arabia. Authorities can benefit from this study results to offer more accessible information about the vaccine importance and safety to the targeted population.

## Introduction

The Coronavirus disease 2019 (COVID-19) pandemic is a global issue that emerged in December 2019 when Wuhan, China, reported the first case [1]. Since then, the number of cases has been increasing and documented in many different countries. As COVID-19 is an infectious disease, vaccination is one of the most successful and effective ways to control the pandemic worldwide [2]. Saudi Arabia has a well strict structured response to controlling the pandemic, providing the required healthcare equipment, and placing testing and vaccination centers in all major regions [3]. However, the logistical challenge is that the government is trying different strategies to convince millions of people of the benefits, safety, and necessity of being vaccinated against COVID-19 [3].

Concerns about any new vaccine are an issue the world faces as the public refuses to take it. On the other hand, vaccines may cause unintended consequences or side effects. These side effects and consequences build a barrier of hesitancy in accepting to be vaccinated with a new vaccine [4].

One of the reasons to refuse the vaccination is a child’s illness, including neurodevelopmental disorders. Many studies gave facts about the rate of parents who discontinue or stopped following their child’s vaccination schedule on purpose after receiving their child’s diagnosis. Up to 50% refusal rate was reported in the United States, besides the misbelief they developed that vaccination caused their child’s neurodevelopmental disorder. In addition, the parents started to refuse to vaccinate their other healthy children [5, 6]. A cross-sectional study conducted in the United States from October 2016 to November 2017, showed a high vaccine hesitancy among parents of children and that they attributed autism spectrum disorder to toxins in vaccines leading them to be concerned about making their child’s disorder worse after taking future vaccine shots [7]. Other studies, in the United States, which was conducted between 1995 and 2015, showed that children with ASD and their younger siblings were under-vaccinated compared with the general population [8, 9]. As far as published, studies in Saudi Arabia explore hesitancy among parents in general [10–12]. On the other hand, this study is putting parents of children with autism and other neurodevelopmental disorder under the spotlight. Hence, vaccination success will rely on public awareness and willingness to be vaccinated, this study can help in making the awareness of the parents a priority and goal for the government.

Under the current circumstances of the COVID-19 pandemic; This study aims to explore the beliefs of parents of children with neurodevelopmental disorders about the COVID-19 vaccine, examine their hesitancy in vaccination, and how certain factors influencing the vaccine decision-making process differ between parents with a child diagnosed with neurodevelopmental disorders and other parents with healthy children.

## Methods

### Study design, participants, and sampling

This cross-sectional study was conducted from August to November 2021 using convenient sampling statistical techniques to quantify the acceptance of COVID-19 vaccination between parents of children with the neurodevelopmental disorder compared to other children and explore their beliefs regarding their vaccination hesitancy. An online survey was distributed to parents in all major Saudi Arabia regions through social media applications. For parents of children with neurodevelopmental disorders, the survey was sent to them via the national centers for neurodevelopmental and behavioral disorders, and specific social media groups that those parents are part of.

Participation in the study was voluntary, and all answers were anonymous. Parents were queried and approached to fill in the questionnaire about their acceptance of COVID-19 vaccination for their children aged 18 years old or under. The questionnaire was completed autonomously by the respondent after obtaining online consent. A sample size of 385 subjects was calculated based on the number of people who are married, divorced, or widow in the Kingdom of Saudi Arabia (KSA) taken from the population census done by the General Authority for Statistics in 2020, which represent Around 9,233,466 people, 33.77% of them are married, 1.27% are divorced, and 0.5% are a widow. Using a confidence level of 95% and a margin error of 0.05, the suitable sample size was calculated by using the Steven equation [13].

Reducing the selection bias for the convenient sample was done in two different ways.

The first one is by using probability sampling along with a convenient sample and diversity. This was done by distributing the questionnaire through social media; among all Saudi social media users, which gave them the same chance and probability to answer the questionnaire, in addition to the diversity of distributing the questionnaire through different applications, different days, and different times a day, and all were done without making a previous judgment about who will be interested in answering the questionnaire and who is not.

The second one was by collecting large data and applying the inclusion and exclusion criteria to them. The collected responses were 400 responses, 381 participants matched the required criteria to fit with the study, and the rest of the (19 participants) were excluded as they did not match the criteria either in the age of children, parents of children with other health issues, or parents of children from other countries.

The questionnaire included 30 questions categorized into specific items: (A) demographic, (B) socioeconomic, and (C) diagnostic information. 10 questions were focused on demographic and socioeconomic data, whilst the rest of them focused on vaccination, neurodevelopmental disorders, and parents’ beliefs. Questions included parents’ age, educational level, marital status, geographic location, employment status, number and age of children, and the diagnosis of neurodevelopmental disorders. To identify their point of view behind their vaccination hesitancy, all respondents were asked about missing any shot of the vaccination schedule on purpose. Parents of children with neurodevelopmental disorders were asked specifically if they have any thoughts or beliefs about a relation between the vaccines and the cause of their child’s diagnosis. The average time of survey administration and the purpose of the study were both provided to the participants. All the methods used in this study were carried out in accordance with relevant guidelines and regulations.

### Translation into Arabic

The questionnaire in this study was first written in Arabic by the investigators themselves then it has been translated into English by professional-level translators. Some questions have been taken from the Parent Attitude about Childhood Vaccines (PACV) validated survey and some modifications have been applied to serve the study purpose [14]. The translated survey went through a back-translation process to confirm that the items matched accurately, were reliable, and were validated. The internal consistency of the Arabic version of the questionnaire was tested using the Cronbach Alpha test giving a score of 0.71 as a reliable and validated questionnaire. The study protocol was reviewed and approved by The Local Committee for Scientific Research Ethics at King Fahad Hospital.

### Statistical analysis

IBM SPSS (Statistical Package for the Social Science) for Windows, 28.0 version, and Microsoft Excel 2016 were used to analyze the research data. The frequency of the variables was calculated to show the exact number and percentage of each item. Chi-square was used as the main test to analyze the data and achieve the goals of this study in exploring the beliefs and willingness of parents of children with neurodevelopmental disorders about the COVID-19 vaccine along with comparing the decision-making process between parents with diagnosed children and parents with healthy children.

## Results

### Survey results

**Fig. 1:**
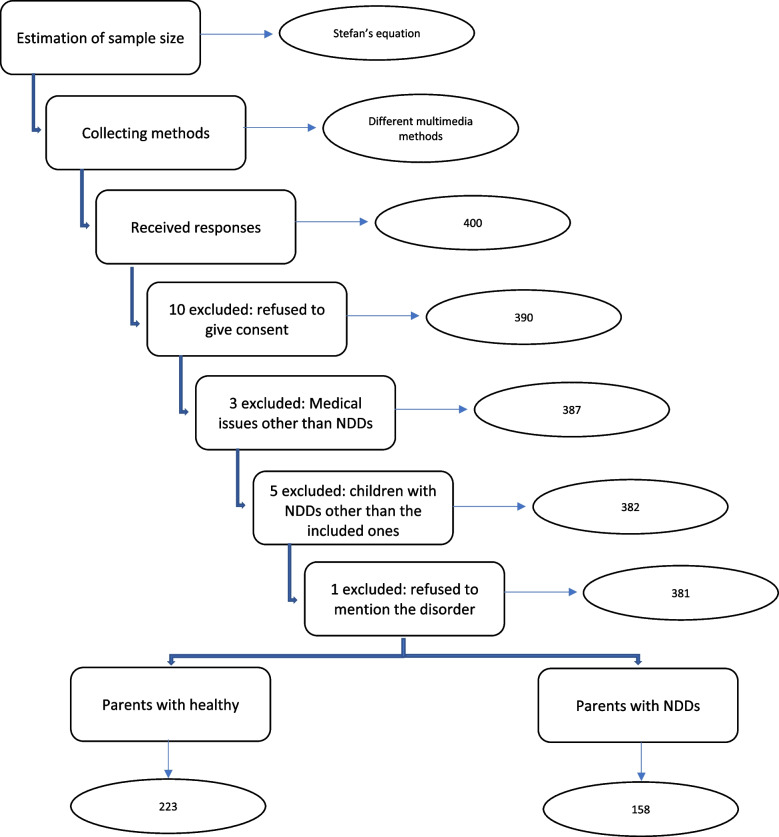
A flowchart representing the study participants

In total, 381 out of 400 completed the online survey and matched the required criteria in the sample simultaneously, with a (95.25%) response rate (Fig. 1).

Table 1 shows the sociodemographic characteristics, including the parents’ age, educational level, marital status, living area, and the number of children. This section includes questions regarding the perception of the COVID-19 pandemic and whether the parents are healthcare workers or have a relative working in a health institute, which represents important factors that could be a reason for the vaccination decision. The majority of the participating parents were above 35 years old, married, had an association or bachelor educational degree, living in the east region area of Saudi Arabia, and raising three children. The data analysis proves that most participants were not healthcare workers (81.9%), but they do have a healthcare relative worker (73.2%).

**Table 1:** Socio-demographic characteristics (*n* = 381)

**Characteristics**	**N**	**%**
**Mother’s age:**		
18–35	161	42.3
Above 35	220	57.7
**Mother’s education level:**		
Middle school or below	19	5.0
High school	90	23.6
Associated or bachelor	225	59.1
Master or above	39	10.2
Uneducated	8	2.1
**Father’s age:**		
18–35	100	26.2
Above 35	281	73.8
**Father’s education level:**		
Middle school or below	45	11.8
High school	95	24.9
Associated or bachelor	177	46.5
Master or above	57	15.0
Uneducated	7	1.8
**Marital status:**		
Married	359	94.2
Separated	12	3.1
Others	10	2.6
**Living area:**		
South	13	3.4
North	13	3.4
Central	54	14.2
West	35	9.2
East	266	69.8
**Number of children:**		
One child	63	16.5
Two children	73	19.2
Three children	245	64.3
**Healthcare worker parents**		
Yes	69	18.1
No	312	81.9
**Relative healthcare worker**		
Yes	279	73.2
No	102	26.8

Table 1 shows the socio-demographic characteristics of the respondents. The majority of the parents were above 35 years of age, married, have an associate or bachelor’s degree, living in the eastern region of Saudi Arabia, raising three children, and were not a healthcare workers but have a relative healthcare worker

One hundred fifty-eight out of 381 participants were parents of children with neurodevelopmental disorders (41.5%). This 41.5% represents the percentage of parents of children with neurodevelopmental disorders that were included in this study sample.

Most of the respondents were parents of children with attention deficit/hyperactivity disorder (ADHD) (41.7%). After that came the diagnosis of autism spectrum disorder (ASD) (34.8%), language and speech disorders (15.1%), Intellectual disability (6.9%), and Motor disorders like developmental coordination disorder (1.2%), making it the least recorded disorder in this study.

Table 2 shows the primary goal of the research by interpreting the vaccination acceptance (32.5%), refusal (12.6%), or hesitation (13.4%) among parents with healthy children compared to the vaccination acceptance, refusal, or hesitation among parents of children with neurodevelopmental disorders (22.3%), (9.7%), (9.4%) respectively. The results also explore the attitudes and beliefs about the previous vaccines and whether they have already missed any vaccine for personal reasons other than health issues such as allergies or illnesses.

**Table 2:** Variables associated to vaccination beliefs and decision-making of COVID-19 vaccination. Diagnosed children (*n* = 158 = 41.5%), Healthy children (*n* = 223 = 58%)

**Variable**	**Diagnosed children**		**Healthy children**	
	**N**	**%**	**N**	**%**
	**158**	**41.5%**	**223**	**58.5%**
**Decision of vaccinating your child**				
Yes	85	22.3	124	32.5
No	37	9.7	48	12.6
Not sure (hesitant)	36	9.4	51	13.4
**Hesitation on a scale**^**a**^				
Not hesitant at all	25	6.6	45	11.8
Not hesitant	8	2.1	11	2.9
Neutral	21	5.5	29	7.6
Hesitant	14	3.7	27	7.1
Extremely hesitant	29	7.6	23	6.0
**“Doctor’s recommendation is an important factor in vaccination decision-making”**				
Strongly disagree	19	5.0	25	6.6
Disagree	9	2.4	11	2.9
Neutral	29	7.6	46	12.1
Agree	13	3.4	20	5.2
Strongly agree	88	23.1	121	31.8
**“It is better for my child to develop immunity by getting sick than to get a vaccine shot”**				
Strongly disagree	67	17.6	93	24.4
Disagree	11	2.9	25	6.6
Neutral	34	8.9	56	14.7
Agree	9	2.4	13	3.4
Strongly agree	37	9.7	36	9.4
**“Vaccine convinces (vaccination method, frequency, distance to vaccination sites, etc.) is an important factor in vaccination decision making”**				
Strongly disagree	38	10.0	36	9.4
Disagree	9	2.4	11	2.9
Neutral	35	9.2	53	13.9
Agree	18	4.7	39	10.2
Strongly agree	58	15.2	84	22.0

Table 2 shows three factors that influence parents’ vaccine-decision-making process regarding COVID-19 vaccination. The majority of parents who would like to vaccinate their children whether they have diagnosed or healthy children, agreed that doctors’ recommendations are an important influence

^a^Not required question

Table 3 represents specific factors that are contributors to the vaccination-decision making process. Parents who think the pandemic is severe show the highest level of acceptance of the COVID-19 vaccine among parents with a neurodevelopmental disorder child. However, parents who have relative healthcare workers show the highest level of acceptance among parents of healthy children.

**Table 3:** The relationship between the decision of vaccinating healthy and diagnosed children, in association with different variables

**Variable**	**Would you vaccinate your child?**							
	Yes	No	Hesitant	***P* value**	Yes	No	Hesitant	***P* value**
	**Diagnosed children**				**Healthy children**			
**Healthcare worker parents**								
Yes	12	5	4	**(0.223)**	29	7	12	**(0.102)**
No	73	32	32		95	41	39	
**Relative healthcare worker**								
Yes	58	24	20	(0.006)**	92	40	45	(0.002)**
No	27	13	16		32	8	6	
**Perception of COVID-19 pandemic**								
Sever	80	22	31	(0.0001)**	112	28	42	(0.0001)**
Exaggerated	5	15	5		12	20	51	
**Deciding not vaccinating the child for reasons other than illness or allergy**								
Yes	8	18	7	(0.0001)**	18	18	12	(0.004)**
No	77	19	29		106	30	39	
**Beliefs of the vaccines as a cause of the child’s neurodevelopmental disorder**^**a**^								
Yes	2	13	1	(0.0001)**	-	-	-	-
No	55	11	19					
I do not know	28	13	16					

Table 3 shows the relationship between the contributing factors and the decision to vaccinate both healthy and diagnosed children. Most of the variables have a significant relationship, therefore the decision of the parent’s decision was influenced by these different variables

**This is the *P*-value indicating the significant relationship between the two variables (< 0.05)

^a^This question was not asked for parents of healthy children

An essay question was asked to parents who were hesitant or refusing vaccination in both groups, those of diagnosed and healthy children, and about the reasons why they refused the vaccine. A total of 79 out of 131 responses were received from both parent groups. Fear of long-term side effects was the most common reason reported by 41 responders out of 64 (64.06%) from parents of healthy children and 38 responders out of 67 (56.71%) from parents of diagnosed children. Another reason reported by parents of younger children in both groups was the child’s age.

### Interpretation of the associated factors and beliefs that influence vaccination decision

When measuring the relationship between the different variables and the vaccination decision, some showed a significant relationship while others did not. The results have shown that having healthy or diagnosed children has a non-significant association with vaccination decision-making as it does not make any difference. In addition, vaccination decisions have neither a relationship to a specific neurodevelopmental disorder nor a relationship with children who have been diagnosed with more than one disorder.

Most of the sociodemographic characteristics had a non-significant relationship with the vaccination decision, with a > 0.05 *p*-value, including educational level and marital status, except parents’ age, which showed a significant relationship with the vaccination decision in both healthy and diagnosed children. There is a non-significant relationship between the healthcare parents’ workers and the decision of vaccinating their healthy or diagnosed child. Surprisingly, statistics have shown a significant relationship between having a healthcare relative worker and the decision of vaccinating their healthy or diagnosed children (*p* = 0.002), (*p* = 0.006), respectively.

The analysis showed a significant relationship between the respondent’s perception of the pandemic and their decision to vaccinate healthy and diagnosed children (*p* = 0.0001).

As shown in Table 2, some variables stand for a valid role in the decision-making. Doctor’s recommendations and vaccine convinces have shown a significant relationship between vaccination decision-making in healthy and diagnosed children (*p* = 0.0001). In multivariate analysis, one way ANOVA test shows a significant relationship between these two factors in both parent groups. Another significant relationship that was revealed by one way ANOVA test is between parents’ decision and not vaccinating the child for reasons other than illness or allergy in both parents’ groups.

In addition, there is a significant relationship between the beliefs of getting immunity by getting sick rather than getting a vaccine shot and the vaccination decision in both healthy and diagnosed children (*p* = 0.0001).

## Discussion

This study is one of the first studies measuring the difference in COVID-19 vaccination acceptance rate between parents of autism and other neurodevelopmental disorders and other parent groups in Saudi Arabia. As vaccination has a crucial role in controlling highly infectious diseases, it is important to study vaccination acceptance rates in the population [2].

**Fig. 2:**
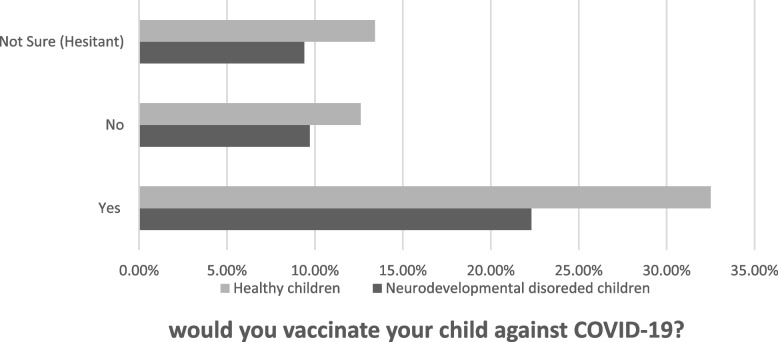
Showing the percentage of acceptance, hesitancy or refusal of COVID-19 vaccination among parents of healthy children vs parents of children with neurodevelopmental disorders

The results suggested that most parents of both diagnosed and healthy children’s groups would vaccinate their children (54.8%), with the highest level of acceptance shown by parents of healthy children (32.5%) as shown in (Fig. 2). Similar to a recent study in Saudi Arabia reported (53.7%) of parents with healthy children were willing to vaccinate their children among 333 respondents [15]. Another study in China reported (72.6%) an acceptance rate of COVID-19 vaccination among the general population [16]. The difference in the acceptance rate is likely related to the different targeted populations, and possibly because the COVID-19 vaccine is considered a new vaccine compared with other vaccines. Unlike the previous studies, which focus on the general population, this study is more specific to target both parents of healthy children and neurodevelopmental disorders children.

Even though the majority of respondents showed a high acceptance rate, If the hesitant parents were added with parents who refused to vaccinate their children to create one group, around 45.14% (the total number (n) 172 out of 381) will be almost equal to the number of parents who accept to vaccinate their children 54.85% (*n* = 209 out of 381) with a small difference between these two groups. These findings play a crucial role in showing the importance of establishing new interventions and campaigns to increase their education about vaccines.

One way ANOVA test was done as a part of the multivariate analysis; shows that parents’ perception about the COVID-19 pandemic as well as not vaccinating the child for reasons other than illness or allergy has a direct relationship with parents’ decision whether to vaccinate or not in both parents groups.

In this study, a demonstration of a correlation between parents’ perception of the COVID-19 pandemic and their decision to vaccinate their children of both groups, is shown in Table 3. Most parents in this study who thought the COVID-19 pandemic was dangerous would vaccinate their children (50.3%). In contrast, most parents who believed that the pandemic was exaggerated would not vaccinate their children (9.1%), and one of the causes was that they thought it was better for their children to develop immunity by getting sick than to get vaccinated, in addition to long term side effects. In another study done in the United Kingdom, addressing parental vaccine hesitancy and childhood influenza vaccine, parents believed that their children should build immunity by getting sick rather than getting vaccinated confirming the existence of this belief [17].

There was no significant relationship between being a parent working in the health field and accepting COVID-19 vaccination, as shown in Table 3. This result could be impacted by the low number of parents working in the health field involved in this study. However, is similar to a study conducted in China, which revealed that Chinese healthcare workers showed relatively low parental acceptability of COVID-19 vaccination for children under the age of 18 years [18]. On the other hand, being a parent with a relative who is a healthcare worker showed a significant relationship with the COVID-19 decision-making process, as these parents were more likely to vaccinate their children. These results are similar to another study done in Turkey, which showed that the vaccine uptake of children with parents who have a healthcare worker, friend, or relative was higher [19].

Parents’ age seemed to affect their vaccination decision. The majority of parents who would vaccinate their children were above 35 years of age for both diagnosed and healthy groups. Similar to the study that has been conducted among parents of children with unspecified health status in the Eastern Mediterranean region, this can be explained by several factors including educational, social, and personal effects [12]. This finding was in contrast with a study conducted in the United Arab Emirates, showing that parental vaccine hesitancy has no significant association with parents’ age [14].

In contrast to the non-significant association between the educational level and the parents’ decision in this study; a study in the United States revealed that the level of parental education is a contributing factor to vaccine hesitancy [20]. Several studies demonstrated that parents with less formal education have greater distrust in the medical community, express more concerns about vaccine safety, and have less belief in the necessity and efficacy of vaccines. Also, it has been found that parents with less than 12 years of education were more likely to report not having enough vaccination information compared with parents with some graduate school education [21–25].

As with many other studies, this study’s results have revealed that parents of both diagnosed and healthy groups who are willing to vaccinate their children were influenced by doctors’ recommendations, as shown in Table 2. Similar results were found in a study in Turkey, showing that physicians had a positive effect on the parents who accepted the vaccine [19]. The role of healthcare providers is crucial to overcome vaccine risks and to benefit from communication with the parents in the matter of influencing their decision of vaccinating their children [26, 27]. Other studies discussing vaccines preventing otitis media and seasonal influenza revealed that parents valued their physicians’ recommendations and are influenced by them [28, 29]. In relation to perception, parents of healthy children who thought that the pandemic was dangerous were affected by doctors’ recommendations and would vaccinate their children. However, parents of healthy children who believed that the pandemic was exaggerated were not affected by these recommendations. According to parents of diagnosed children, there was no relation between doctor’s recommendations and parents’ perceptions.

More than half of parents (60%) from both groups who refused the new COVID-19 vaccine expressed their fear about the safety and long-term side effects of the vaccine on their children, especially since this vaccine is still under trial for this age group and there are not enough studies about it. Another possible reason for the high rejection rate is that many parents, particularly those with affected children, believe that vaccines cause neurodevelopmental disorders in their children and since COVID-19 is a newly discovered virus, many people believe it never existed in the first place and that it is a conspiracy to destroy humanity. It could also be linked to the fact that many parents are waiting for some time before deciding to vaccinate their child since they want to make sure it is devoid of side effects first. A national survey involving more than 11,000 parents in the USA identified that parents who delayed and refused vaccines were more likely to have vaccine safety concerns and perceived fewer benefits associated with vaccines [30]. Similar results have been found in another study which reported that vaccine safety issues were one of the parents’ top concerns [14].

## Recommendations

As healthcare providers’ recommendations significantly impact parents’ decisions from both groups, national campaigns involving pediatrics providing educational interventions for parents, especially vaccine-hesitant parents, will considerably improve the acceptance rate. Additional information about the safety and efficacy of the COVID-19 vaccine on children through trusted channels besides web-based vaccine information with social media applications targeting especially the younger parents will help to increase the acceptance rate and build trust; hence the young parents showed lower acceptance rate [31]. Another possible effective strategy to increase the acceptance rate is to conduct in-person meetings with parents of unvaccinated children to relieve their fears and to correct the misconceptions. A reminder about the role of vaccines in overcoming the previous pandemics and the expansion of vaccine centers with additional facilities can help as well.

## Limitations

This study has a few limitations, one of them was that healthcare worker parents who refused the vaccine were considerably low in number compared to parents with a relative in the health field. Another limitation was the participants in the Eastern region were overly represented in this study compared to other regions in the Kingdom of Saudi Arabia (KSA). Also, this study is not having the child’s age as a factor that may influence the parents’ decision hence parents of children younger than 12 years may have different decision than parents of children older than 12 years. The time conduction of this study is a limitation as well. There was no social restriction on children who aren’t vaccinated. In addition, parents’ decisions could be influenced by other factors which were not asked in the distributed questionnaire and not explored in this study indeed. Lastly, the sample size of this study is relatively small, future studies should focus on having a larger sample size and choose different study design as the convenient design has many biases.

## Conclusion

About 54.8% of parents of both groups showed a willingness to vaccinate their children with the COVID-19 vaccine. Parents’ age and beliefs, doctor’s recommendations, vaccines convince, having a health care worker relative, and parents’ perception of the pandemic, all of which were factors measured in this study and have affected parents’ decision-making process. However, the remaining participants, representing 45.2%, refused or were uncertain about vaccinating their children. The fear of the side effects whether long or short-term side effects was the most important reason.

## Data Availability

The dataset used and/or analyzed during the current study is not publicly available due to the confidentiality commitment but is available from the corresponding author on reasonable request.

## Abbreviations

ASD: Autism Spectrum Disorder
KSA: Kingdom of Saudi Arabia
PACV: Parent Attitude Childhood Vaccines
SPSS: Statistical Package for the Social Science
ADHD: Attention-deficit/hyperactivity disorder
