# Correction to: Acceptance of COVID-19 vaccination among parents of children with autism and other neurodevelopmental disorders in Saudi Arabia: a cross-sectional study

**DOI:** 10.1186/s12889-023-16351-x

**Published:** 2023-09-28

**Authors:** Ali Jawad Al Saad, Mohammed Ghadeer Alhassan, Maryam Saleh, Fatimah Fathi Alqattan, Fatimah Ali Aleisa, Hawra Wasel Abdulmohsen

**Affiliations:** King Faisal University, Alahsa, Saudi Arabia

**Correction to**: ***BMC Public Health *23, 1235 (2023)**

10.1186/s12889-023-16127-3

The original publication of this article contained 3 errors:

1. The author name “**Fatimah Ali Alessa**” should have been “**Fatimah Ali Aleisa**”.
2. The author name “**Hawra Wasel Alabdulmohsen**” should have been “**Hawra Wasel Abdulmohsen**”.
3. The funding number “**Project No.545**” should be “**Project No.3694** “.

The original article has been updated to correct these errors.

